# Functionalized Cortical Bone‐Inspired Composites Adapt to the Mechanical and Biological Properties of the Edentulous Area to Resist Fretting Wear

**DOI:** 10.1002/advs.202207255

**Published:** 2023-02-12

**Authors:** ZhongYi Wang, QianRong Xiang, Xin Tan, YaDong Zhang, HaoQi Zhu, Jian Pu, JiKui Sun, ManLin Sun, YingKai Wang, Qiang Wei, HaiYang Yu

**Affiliations:** ^1^ State Key Laboratory of Oral Diseases National Clinical Research Center for Oral Diseases West China Hospital of Stomatology Sichuan University Chengdu Sichuan 610041 China; ^2^ Chongqing Key Laboratory of Oral Diseases and Biomedical Sciences College of Stomatology Chongqing Medical University Chongqing 400016 China; ^3^ Research and Development Department Zhejiang PEKK‐X Advanced Materials Technology Co., Ltd. Shaoxing Zhejiang 312000 China; ^4^ Department of Physics City University of Hong Kong Hong Kong Special Administrative Region of the People's Republic of China Kowloon 999077 China; ^5^ School of Mechanical Engineering Southwest Jiaotong University Chengdu Sichuan 610031 China; ^6^ College of Polymer Science and Engineering State Key Laboratory of Polymer Materials and Engineering Sichuan University Chengdu Sichuan 610065 China

**Keywords:** biological–mechanical adaptivity, dentition defects, fretting wear, implant–bone interface, zirconia

## Abstract

Dental implants with long‐term success of osseointegration have always been the goal, however, difficulties exist. The accumulation of fretting damage at the implant–bone interface often gets overlooked. Commonly used titanium is approximately 7‐fold harder and stiffer than cortical bone. Stress shielding caused by the mismatching of the elastic modulus aggravates fretting at the interface, which is accompanied by the risk of the formation of proinflammatory metal debris and implant loosening. Thus, the authors explore functionalized cortical bone‐inspired composites (FCBIC) with a hierarchical structure at multiple scales, that exhibit good mechanical and biological adaptivity with cortical bone. The design is inspired by nature, combining brittle minerals with organic molecules to maintain machinability, which helps to acquire excellent energy‐dissipating capability. It therefore has the comparable hardness and elastic modulus, strength, and elastic‐plastic deformation to cortical bone. Meanwhile, this cortical bone analogy exhibits excellent osteoinduction and osseointegration abilities. These two properties also facilitate each other to resist fretting wear, and therefore improve the success rate of implantation. Based on these results, the biological–mechanical co‐operation coefficient is proposed to describe the coupling between these two factors for designing the optimized dental implants.

## Graphical abstract

The accumulation of fretting damage at the implant–bone interface often gets overlooked. A functionalized cortical bone‐inspired composite with hierarchical structures at multiple scales, possessing a good co‐operation of mechanical and biological adaptivity with cortical bone, is exploited. The two properties of this implant also facilitate each other to resist fretting wear, and therefore improve the success rate of implantation.

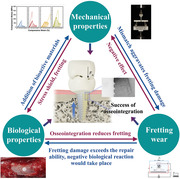

## 1 Introduction

Oral health is essential to a good quality of life.^[^ ^1^ ^]^ In the 4th Chinese oral health survey, 84.4% of adults had dentition defects and 1.8% had edentulism. Dental implant restoration with high clinical efficacy has become the main restoration technique for dentition defects.^[^ ^2^ ^]^ Titanium and related alloys with high osseointegration rates are preferred in clinical therapeutics, especially low elastic modulus alloys. However, the grey color may shine through the thin mucosa resulting in an aesthetic issue.^[^ ^3^ ^]^ Metal particles released by corrosion have been shown to lead to peri‐implantitis.^[^ ^4^ ^]^ Therefore, metal‐free implants with tooth‐like coloration, such as yttrium‐stabilized tetragonal zirconia (Y‐TZP), polyetheretherketone (PEEK), and polyetherketoneketone (PEKK), have become potential alternatives to titanium in implants and are receiving considerable attention.^[^ ^5^ ^]^ However, their application in clinical practice is challenging, due to the practical obstacles associated with bioinertia and the difficulty in obtaining bone‐matching mechanical properties.

Once a dental implant is placed into the edentulous area, biological effects in the bone–implant interface come into play.^[^ ^6^ ^]^ A sequence of molecular and cellular reactions determines acceptance or rejection of the replacement. These reactions are correlated with the chemical and physical properties of the implant surface, such as roughness, wettability, biocompatibility, and corrosion resistance.^[^ ^3^, ^7^ ^]^ Besidesbiological compatibility and bioactivity, the mechanical adaptivity of implants with cortical bone is another major consideration, as a poor compatibility may cause excessive fretting wear of implants triggering the failure.^[^ ^8^ ^]^ After osseointegration in an edentulous area, dental implants are subjected to cyclic occlusal forces. During chewing, the forces spread and dissipate from the implant system to the surrounding alveolar bone; in this process, fretting inevitably occurs at the dental implant‐bone interface, leading to cumulative damage.^[^ ^9^ ^]^ The hardness and elastic modulus of human cortical bone are approximately 0.5 and 15 GPa, respectively. The most widely used titanium implant is approximately 7‐fold harder and stiffer than cortical bone, while ivory‐colored 3 mol% Y‐TZP (3Y‐TZP) is ≈30 times harder and ≈11 times stiffer than cortical bone.^[^ ^10^ ^]^ Similar t tribology in engineering, osseointegrated dental implants and cortical bones form a classical fretting friction pair (<100 µm). The mismatch in mechanical properties between the bone and implant aggravates fretting damage, especially overloading, thereby disrupting the tight contact of the interface. Debris from titanium wear may aggravate the pathology of peri‐implant bone loss.^[^ ^11^ ^]^ Thus, for one thing, an ideal dental implant material should possess high mechanical adaptivity with a comparable hardness and elastic modulus to cortical bone in order to effectively reduce fretting wear while alleviating the stress shielding related to marginal bone loss.^[^ ^12^ ^]^ In addition, the long‐term success of osseointegration enables mitigating fretting wear. Therefore, it is essential to consider biological–mechanical adaptivity between the implant and cortical bone.

Recent studies focusing on increasing the surface roughness and wettability and introducing more —OH groups offer feasible solutions for inducing surface bioactivation.^[^ ^13^ ^]^ For example, acid‐etched 3Y‐TZP implants exhibited better bone–implant contact (BIC) values compared with titanium implants.^[^ ^14^ ^]^ Ultraviolet (UV) light and oxygen plasma treatments can increase the biocompatibility of titanium, zirconia, and PEEK surfaces.^[^ ^15^ ^]^ However, such surface modification can hardly realize double matching of biological–mechanical adaptivity. We learn from nature by looking into the characteristics of cortical bone, which exhibits a hierarchical structure, and serves in overall lifecycle. It comprises ≈65 wt% apatite crystals and ≈25 wt% organic matter (containing ≈90 wt% type I collagen) along with water, and are organized from nano‐, micro‐, to macro‐scales. The minerals are stiff and strong, while the organics contribute to bone toughness and ductility.^[^ ^16^ ^]^ The combination of inorganic and organic matter in biomimetic structures confers high yield strength and fracture toughness and may, in principle, offer new promising alternatives to human organs.^[^ ^17^ ^]^

In this study, we take advantage of the design of cortical bone‐inspired composites (CBIC) to achieve bone‐matching mechanical properties and good osteoinductive effect at the same time. The biological–mechanical co‐operation enhances the osseointegration and resists fretting wear. We used freeze‐casting technology to construct 3Y‐TZP scaffolds with ordered macro‐pores to provide space for incorporating a soft phase.^[^ ^18^ ^]^ Commercially available PEEK and PEKK, renowned for industrial applications, are members of the FDA‐approved, high‐performance (excellent fracture resistance, shock absorption, and stress distribution) polyaryletherketone polymer family.^[^ ^19^ ^]^ The successful combination of 3Y‐TZP and PEEK/PEKK tend to realize our blueprint. PEKK with more ketone groups can facilitate bioactivation and increase its polarity and backbone rigidity. It also exhibits a higher glass transition temperature and melting point and better mechanical, wear, and osteointegration properties than PEEK.^[^ ^20^ ^]^ PEKK produces also have a lower inflammatory response than synthetic materials such as polymethylmethacrylate.^[^ ^21^ ^]^ Thus, we use PEKK as an organic component, and the novel CBIC with hierarchical structures was toughened by the in situ polymerization of PEKK. To obtain a bioactive surface, the CBIC was functionalized by immersion in 30% H_2_O_2_ and UV irradiation.^[^ ^13^, ^22^ ^]^ The biological–mechanical adaptivity to the cortical bone of the functionalized CBIC (FCBIC) and its fretting wear resistance were comprehensively evaluated and theoretically analyzed. Such damage‐tolerant and bioactive composites with improved and novel properties transcend the benchmark of the commonly used implants.

## 2 Result and Discussion

### 2.1 Construction of Biomimetic Microstructures

**Figure 1:**
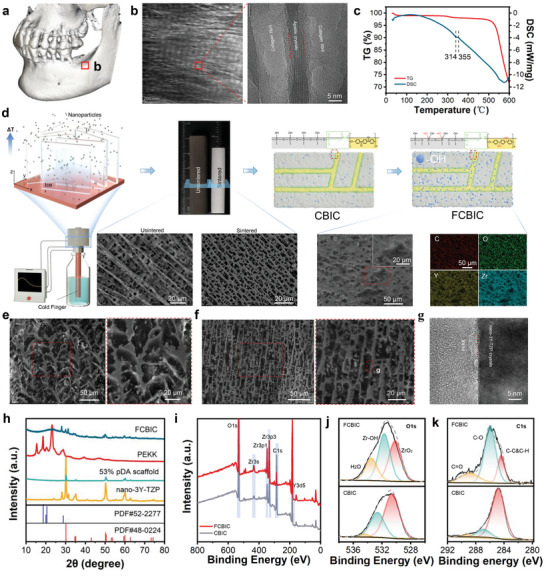
Cortical bone and the synthesis of bioinspired composites. a) CBCT reconstruction of the jaw with an edentulous area. b) Transmission electron microscopy (TEM) imaging of the cortical bone in an edentulous area. c). Combined thermogravimetric analysis (TGA)/differential thermal calorimetry (DSC) curves of polyetherketoneketone (PEKK). d) Schematic illustration of the functionalized cortical bone‐inspired composites (FCBIC) fabrication process; the corresponding cross‐sectional scanning electron microscope–energy dispersive spectroscopy (SEM–EDS) images of 53 wt% pDA‐nano‐3Y‐TZP scaffolds, and FCBIC. SEM images of the sagittal plane of e) 53 wt% nano‐3Y‐TZP/PEKK composites (nano‐3Y‐TZP/PEKK) and f) FCBIC. g) TEM image of FCBIC. h) X‐ray diffraction (XRD) patterns of FCBIC, PEKK, 53 wt% pDA‐nano‐3Y‐TZP scaffolds, and nano‐3Y‐TZP particles. i) X‐ray photoelectron spectroscopy (XPS) analysis of FCBIC and CBIC. j) O1s and k) C1s high‐resolution spectra of CBIC and FCBIC.

To reduce the brittleness of the ceramics without compromising strength, we explored natural products that are tough (e.g., bone, wood, and nacre). According to darkfield images of ion‐ milled sections of cortical bone, we observed oriented apatite crystals, characterized by scatter electrons from a specific lattice plane, between collagen fibrils. Collagen fibrils are closely assembled with lamellar apatite crystals and contribute to cortical bone strength and toughness (**Figure** **1**a,b).^[^ ^16c^ ^]^ Drawing inspiration from the hierarchical architecture of cortical bone, lamellar structured ceramic scaffolds with different mineral contents were created using environmentally friendly freeze‐casting method. A copper cold finger immersed in liquid nitrogen can create a vertical temperature gradient (Δ*T*) to force the vertical growth of ice crystals (Figure 1d). Nanoscale 3Y‐TZP (nano‐3Y‐TZP) powders were employed to improve the grain boundary diffusion and promote the mechanical properties of scaffolds.^[^ ^23^ ^]^

Unsintered 53 wt% nano‐3Y‐TZP scaffolds exhibit obviously uneven lamella thickness and more lateral branches than scaffolds without particles (Figure S1, Supporting Information). These phenomena indicate that nano‐3Y‐TZP tend to agglomerate via strong Van der Waals attraction, thereby hindering homogeneous dispersion in the aqueous slurry (Figure S2a–d, Supporting Information). We utilized the catechol group of dopamine for cross‐linking to form a polydopamine (pDA) film that strongly adheres onto the surface of nano‐3Y‐TZP.^[^ ^24^ ^]^ The pDA‐modified nano‐3Y‐TZP (pDA‐nano‐3Y‐TZP) with a 3% grafting ratio can decrease the viscosity of slurry and tendency to agglomerate by enhancing the interfacial compatibility of nano‐3Y‐TZP in the mobile phase, and improving the uniformity of the dispersion (Figure S2a–d, Supporting Information). In theory, pDA‐nano‐3Y‐TZP slurry has lower ice nucleation resistance and a faster nucleation rate vertically, consequently generating more ice nuclei in a short time.^[^ ^25^ ^]^ In a limited space, these result in more ice lamellae, later substituted by pores, and thinner ceramics lamellae (Figure S3 and Table S1, Supporting Information). We used pDA‐nano‐3Y‐TZP to form pDA‐nano‐3Y‐TZP scaffolds, characterized by regular and thick lamellae in the unsintered state and a thinner lamella and smaller pore width after sintering (Figure 1d). According to the horizontal cross‐sections of the scaffolds, ceramic lamellae are vertically aligned and are located randomly in the horizontal direction. This may be because the direction of ice crystal nuclei is randomly parallel to Δ*T* (Figure S3, Supporting Information). Subsequently, sintered ceramic scaffolds were grafted by *γ*‐MPS and toughened using in situ PEKK polymerization between lamellae (Figure 1d). According to the thermogravimetric analysis and differential thermal calorimetry analysis of PEKK, the composites were heated to 380 °C to enhance PEKK filling between lamella ceramics (Figure 1c). Following in situ polymerization, PEKK molecules polymerize among the scaffolds to create a brick‐and‐mortar composite.^[^ ^7^ ^]^ Next, the surface of the composite was functionalized to achieve biomechanical advantages. Herein, the CBIC are 53 wt% pDA‐nano‐3Y‐TZP composites and functionalized CBIC (FCBIC) are obtained from 30% H_2_O_2_ and UV irradiation. To explore the role of microstructure related to pDA‐modification, we also produced a benchmark 53 wt% nano‐3Y‐TZP‐based composites, namely nano‐3Y‐TZP/PEKK, without pDA‐modification. The ceramic phase in both nano‐3Y‐TZP/PEKK and FCBIC are well bonded with PEKK, and the thicknesses of the ceramic lamella and PEKK phase are respectively ≈6.7 and ≈7.9 µm in FCBIC, respectively (Figure 1d–f and Table S1, Supporting Information). The higher‐magnification transmission electron microscopy images revealed that the ceramic lamella and PEKK phase are closely connected in FCBIC (Figure 1g).

The phase constitution of FCBIC was characterized using XRD and compared with those of nanoparticles, sintered scaffolds, and PEKK. Figure 1i–k shows the X‐ray photoelectron spectroscopy (XPS) survey spectra for CBIC and FCBIC, with the increased peak intensity ratios of O in the FCBIC surface reflecting an oxidized surface. The increase in C—O bonding intensity and the decrease in C=O bonding intensity coincide to demonstrate that PEKK is capable of its own ring‐opening hydrogenation after UV irradiation. The XPS and XRD analyses indicated that the PEKK and FCBIC composites were synthesized successfully.

### 2.2 Mechanical Response and Surface Properties

**Figure 2:**
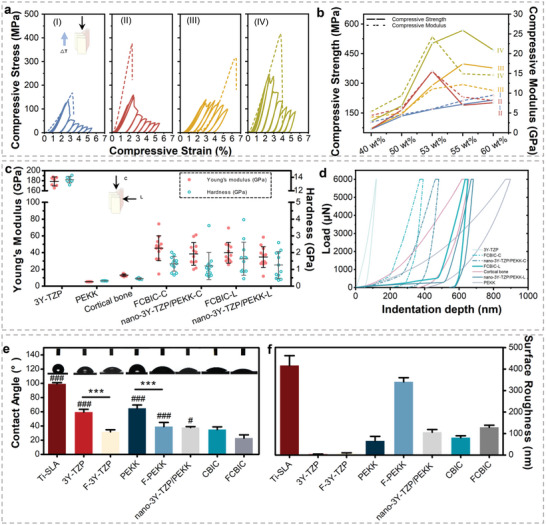
Mechanical and surface properties. a) Compressive stress–strain curves of (I) 53 wt% nano‐3Y‐TZP scaffolds, (II) 53 wt% nano‐3Y‐TZP/PEKK composites (nano‐3Y‐TZP/PEKK), (III) 53 wt% pDA‐nano‐3Y‐TZP scaffolds, and (IV) functionalized cortical bone‐inspired composites (FCBIC). b) Compressive strength and modulus of the scaffolds and composites: (I) nano‐3Y‐TZP scaffold, (II) pDA‐nano‐3Y‐TZP scaffold, (III) nano‐3Y‐TZP/PEKK composites, and (IV) pDA‐nano‐3Y‐TZP/PEKK composites. c) Young's modulus and hardness and d) load–displacement curves of nano‐3Y‐TZP/PEKK and FCBIC, along the longitudinal (L) and cross‐sectional (C) directions, compared with 3Y‐TZP, PEKK, and cortical bone (*n* = 12). e) Water contact angle (WCA) and corresponding photos of the droplets on the surfaces of the materials. ***p < 0.001. ^#^ *p* < 0.05, ^##^ *p* < 0.01, ^###^ *p* < 0.001 compared with FCBIC (*n* = 3). f) Surface roughness of the materials (*n* = 5).

The mechanical response of FCBIC, designed with densified scaffolds and thinner lamellae, was evaluated. 3Y‐TZP and PEKK, the two components, have been proven chemically stable in the oral environment and dentistry field.^[^ ^5^, ^26^ ^]^ Thus, we analyzed the compressive resistance of FCBIC and its scaffolds, compared with nano‐3Y‐TZP/PEKK and its scaffolds in an indoor environment. Monotonic and cyclic compression tests were conducted to simulate chewing. The typical stress–strain curves reflect their compressive resistance, stress resistance, and fatigue performance (**Figure** **2**a). The brittle ceramic scaffolds exhibit lower compressive strength than cortical bone (≈250 MPa) and acquire greater plasticity following PEKK stuffing.^[^ ^27^ ^]^ The compressive strength of FCBIC is 500.27 ± 85.17 MPa, which was higher than that of nano‐3Y‐TZP/PEKK (362.15 ± 51.56 MPa). Given the advantages of the organic component, the plastic deformation of nano‐3Y‐TZP/PEKK surpasses 2.4%, while that of FCBIC surpasses it by up to 2.5%. In addition, FCBIC have more stable hysteresis stress–strain loops under cyclic loading–unloading conditions, indicating that its viscoelastic and plastic properties absorb and dissipate the imposed mechanical energy. We also evaluate the compressive strength and modulus of scaffolds and composites with different mineral contents, in which FCBIC (24.16 ± 0.99 GPa compressive modulus) is comparable to cortical bone of the edentulous area (≈20 GPa) (Figure 2b).^[^ ^27^ ^]^ Considering the anisotropy of composites, we acquired Young's modulus and hardness along longitudinal (L) and cross‐sectional (C) directions. FCBIC‐L directly contact with cortical bone, and has an approximate Young's modulus of 39.80 ± 12.40 GPa, while FCBIC‐C has a higher modulus of 45.34 ± 14.85 GPa.^[^ ^27^ ^]^ FCBIC‐L are built with a hardness of 1.63 ± 0.99 GPa, and FCBIC‐C are 1.33 ± 0.44 GPa (Figure 2c,d).

Surface properties are generally affected by surface chemistry modification.^[^ ^28^ ^]^ The wettability of materials is reflected by hydrophilicity, which was evaluated using the sessile drop method. The functionalized treatment can increase the water contact angle (WCA) of all materials because of the increase in —OH groups. The WCA of FCBIC is 23.03 ± 7.76°, indicating strong hydrophilicity (Figure 2e).^[^ ^13c^ ^]^ The roughness of materials was tested using a 3D surface profilometer. The sandblasted, large‐grit, acid‐etched Ti (Ti‐SLA) has the roughest surface (499.40 ± 55.47 nm), while that of 3Y‐TZP is the smoothest (5.23 ± 1.27 nm). FCBIC and nano‐3Y‐TZP/PEKK have average roughness values of 155.40 ± 11.42 and 127.30 ± 15.40 nm respectively (Figure 2f).

**Figure 3:**
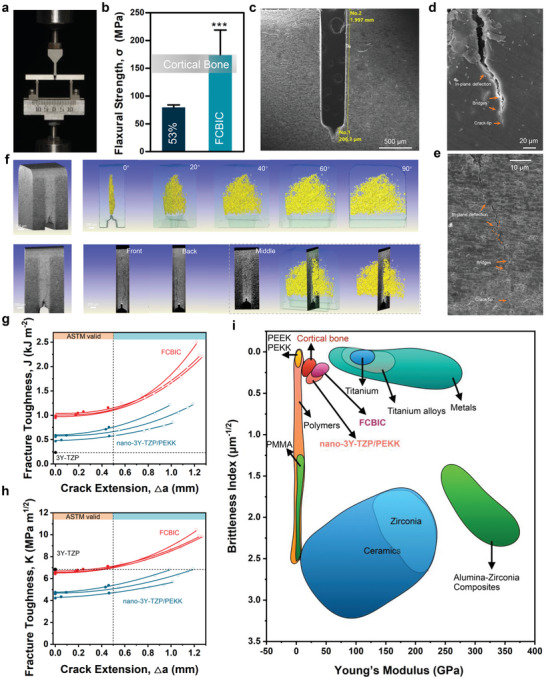
Flexural strength and fracture toughness of 53 wt% nano‐3Y‐TZP/PEKK composites (nano‐3Y‐TZP/PEKK) and functionalized cortical bone‐inspired composites (FCBIC). a) The three‐point bending test condition. b) The flexural strength of nano‐3Y‐TZP/PEKK and FCBIC compared to cortical bone (*n* = 3). c) Scanning electron microscope (SEM) image of UV‐notches on FCBIC. SEM image of crack propagation in d) cortical bone and e) FCBIC. f) X‐ray tomography (XRT) renderings of crack propagation in FCBIC. g) *J*‐integral, and h) *K*‐based fracture toughness with crack extension (*n* = 3). i) Young's modulus and brittleness index for FCBIC, compared with implant materials.

Flexural strength and toughness were tested using three‐point bending (**Figure** **3**a). The flexural strength of FCBIC is 171.10 ± 45.10 MPa, which is similar to that of cortical bone (Figure 3b).^[^ ^29^ ^]^ To analyze the flexural toughness and related crack‐resistance (R‐curve) behavior of composites, we prepared a V‐notch on the specimens to observe crack propagation from the sharpened notch tip using in situ scanning electron microscopy (SEM) (Figure 3c). In situ SEM results clearly revealed the torturous cracking path in FCBIC and cortical bone. After passing through the organic phase, the crack tended to deflect along the organic–inorganic interface, resembling the crack deflection in the cement lines of cortical bone, resulting in a lower local driving force at the crack tip (Figure 3d,e).^[^ ^30^ ^]^ For FCBIC, the organic phase plays a role in improving the crack deflection by maximizing the contribution of organic–inorganic interfaces, and increasing energy‐dissipating capability.^[^ ^17^, ^31^ ^]^ 3D X‐ray tomography images with different rotation angles further showed the complex morphology of the crack in 3D space with the cracking paths continuously deflected (Figure 3f). When crack deflection and crack bridging occur, higher applied stress is required to maintain further crack growth, which is manifested as increased toughness. Dopamine modification leading to specific microstructure affected crack propagation behavior by affecting the composition of the organic and inorganic phases, which resulted in the superior fracture toughness of FCBIC over that of nano‐3Y‐TZP/PEKK. In additional, the difference in Young's modulus between organic and inorganic phases leads to abrupt variation of Young's modulus when the crack extends to the organic–inorganic interface, which is one of the proven mechanisms to reduce the driving force for crack propagation in lamellar structures.^[^ ^32^ ^]^

The *J*‐integral and equivalent *K*‐based fracture toughness values of nano‐3Y‐TZP/PEKK and FCBIC are shown in Figure 3g,h. Similar to the fracture behavior of cortical bone, the resistance to crack propagation increased with crack propagation, which was reflected by a steep rising R‐curve behavior.^[^ ^33^ ^]^ The critical *J*‐integral fracture toughness (*J*_IC_) of nano‐3Y‐TZP/PEKK and FCBIC were ≈0.68 and ≈1.12 kJ m^−2^, respectively, which were significantly higher than that of 3Y‐TZP but lower than cortical bone. The equivalent *K*‐based fracture toughness (*K*_JIC_) of nano‐3Y‐TZP/PEKK and FCBIC are ≈4.86 and 6.68 MPa m^1/2^, respectively. The critical crack extension limitation of 0.50 mm was in strict accordance with the American Society for Testing Material Standard E‐1820.^[^ ^34^ ^]^ The PEKK component did enhance the fracture toughness of 3Y‐TZP.

The brittleness index, as calculated by the ratio of hardness to crack‐initiation fracture toughness (*K*_IC_), can directly evaluate on the machinability of dental materials.^[^ ^35^ ^]^ High fracture toughness and proper hardness result in a low brittleness index, which signifies better machinability, and ease of industrial production. Genenally, the design of dental materials and thier strength, and toughness are usually mutually exclusive.^[^ ^36^ ^]^ We comprehensively evaluated the brittleness index and Young's modulus of FCBIC and other dental implant materials (Figure 3i). FCBIC, designed with a layered structure and filled with an organic phase, exhibit sufficient strength, with Young's modulus and a brittleness index comparable to those of cortical bone. However, the toughness of natural cortical bone is difficult to surpass, therefore more research studies are needed to achieve on the ideal fracture toughness.

### 2.3 Adhesive and Osteoinductive Ability of MSCs on Materials Mediated by Cellular Mechanosensing

**Figure 4:**
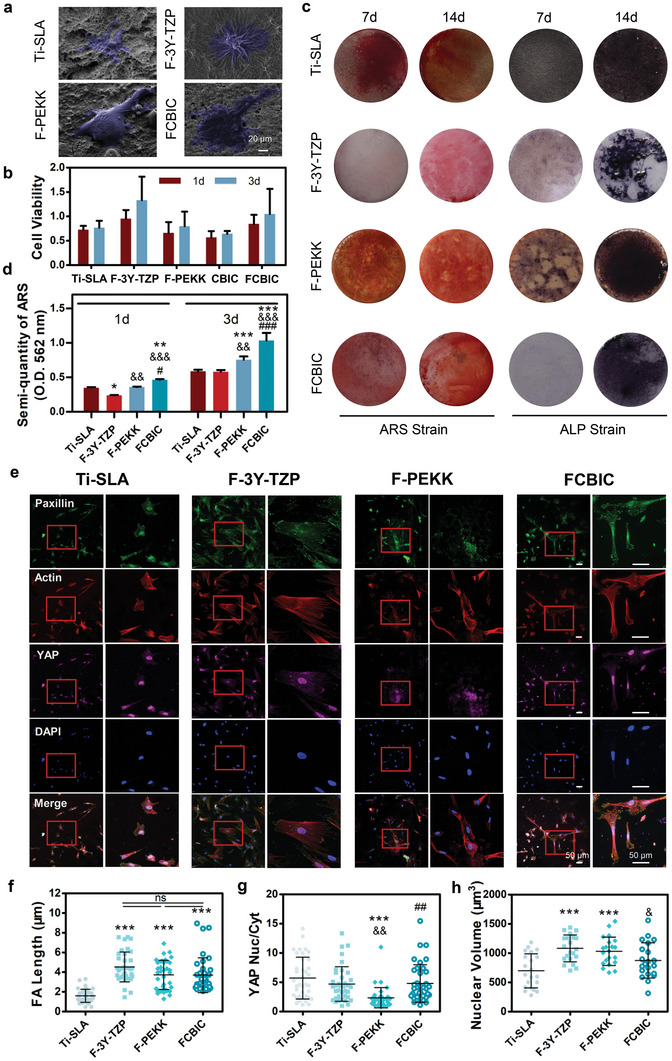
Adhesive and osteoinductive ability of MSCs on materials mediated by cellular mechanosensing. a) Representative SEM images of adhered human umbilical cord Wharton's jelly‐derived mesenchymal stem cells (hWJ‐MSCs) on the functionalized materials. b) hWJ‐MSC viability on materials (*n* = 4). c) Extracellular calcium deposition and alkaline phosphatase (ALP) activity at 7 and 14 days were visualized by Alizarin Red S (ARS) and ALP staining, respectively. d) Quantitative analysis of the extracellular calcium matrix (*n* = 3). e) Representative fluorescent images of paxillin, actin, yes‐associated protein (YAP), and DAPI, captured in hWJ‐MSCs, co‐cultured on the materials for 24 h. f) The length of focal adhesion (FA) of hWJ‐MSCs, as indicated by paxillin immunostaining (*n* = 37). g) Radiometric analysis of the nucleus location of YAP by nucleus/cytoplasmic fluorescence intensity ratio (*n* = 37). h) Nuclear volume (*n* = 37). **p < 0.01, ***p < 0.001 versus sandblasted, large‐grit, acid‐etched Ti (Ti‐SLA); ^&^ *p* < 0.05, ^&&^ *p* < 0.01, ^&&&^ *p* < 0.001 versus F‐3Y‐TZP; ^##^ *p* < 0.01, ^###^ *p* < 0.001 versus F‐PEKK.

Materials with unique surficial physiochemical properties and nano‐topography may influence human umbilical cord Wharton's jelly‐derived mesenchymal stem cells (hWJ‐MSCs)‐surface interactions and biocompatibility, as presented by different cell morphology, viability, and filopodia. The hWJ‐MSCs on FCBIC exhibit the largest spreading area, abundant filopodia (tightly cross‐linked long bundles of actin filaments), sensing the surface (**Figure** **4**a).^[^ ^37^ ^]^ The cell viability of FCBIC is comparable to that of conventional materials (Figure 4b). To visualize the osteoinductive effect of FCBIC on hWJ‐MSCs, the cells were co‐cultured with the materials for 7 and 14 days, followed by Alizarin Red S and alkaline phosphatase (ALP) straining. Increased deposition of mineralized nodules are observed in the FCBIC groups, with semi‐quantitative analysis concomitantly evaluated, indicating that more hWJ‐MSCs differentiate into osteoblasts. Cells in FCBIC group shows darker and denser blue staining on day 14 than conventional materials, indicating the higher ALP activity and osteoblast differentiation (Figure 4c,d). PEKK also shows unique osteoinductivity, consistent with previous reports.^[^ ^38^ ^]^ Thus, we inferred that FCBIC possess a desirable in vitro osteoinductive effect.

The osteogenic differentiation of the cells is associated with the material surface properties. Previous investigations have proven that materials with 150–450 nm roughness, especially approximately 278 nm, have the best cell adhesion.^[^ ^37^, ^39^ ^]^ Notely, FCBIC has an optimal roughness of 155.40 nm in these groups. High hydrophilicity is known to promote an environment conducive for bone formation. Serum can rapidly spread on surfaces with suitable hydrophilicity, providing an overall coating of bioactive factors, and a favorable surface for cell adhesion and differentiation.^[^ ^40^ ^]^ In our study, FCBIC have the highest hydrophilicity of 23.03°. However, it is inappropriate to ascribe the ultimate effect to a single factor, such as roughness, rigidity, or a chemical factor.^[^ ^41^ ^]^ In general, both biochemical signals and mechanical and topographic cues can affect cell adhesive behaviors via the extracellular matrix, which can be explored by focal adhesion (FA) and the alignment of cyto‐ and nucleoskeletal elements. Favorable surface properties of materials may trigger FA formation to sense the nano‐ or micro‐topography, and reinforce cellular mechanosensing. In parallel, corresponding signaling cascades are initiated, and cellular force is conducted from the cytoskeleton and the integrins to the nucleus.^[^ ^42^ ^]^ Previous studies have shown that long actin fibers, an adequate area of FAs, and high nuclear tension are favorable to the osteogenic differentiation of MSCs.^[^ ^37^ ^]^ Thus, the inherent correlation needs to be determined between the osteoinductive effect of the surface of materials, and corresponding cell adhesion behavior needs to be determined.

Herein, after attaching on materials, hWJ‐MSCs acquire traction force and transfer it from actomyosin to the integrins. As shown in Figure 4e,f, all groups have enough force to generate FAs, as verified by immunofluorescent staining of paxillin. FA length in FCBIC is comparable to F‐3Y‐TZP and F‐PEKK groups, while Ti‐SLA has the shortest FAs. Actin stress fibers attach FAs to grow, cross‐link, and organize the actin cytoskeleton. FCBIC has the longest bundles of actin stress fibers, enabling the nucleus to acquire more traction force (Figure 4e). Yes‐associated protein (YAP) is one of the important transcriptional regulators of mechanical cues, as well as osteogenesis pathways.^[^ ^43^ ^]^ We calculate the YAP nuclear/cytoplasmic accumulation in adhesive cells, and note that FCBIC and Ti‐SLA has similarly high values of 4.80 and 5.70, respectively (Figure 4g). With more force conduction into the nucleus, both FCBIC and Ti‐SLA have low nuclear volume, 875 and 697.80 µm^3^, respectively, indicating the high nuclear tension and accompanying osteogenesis effect (Figure 4h). Thus, compared with commonly used dental implant materials, FCBIC has superior in vitro osteoinduction ability compared to Ti‐SLA, which can be ascribe to the longer FA length. The nucleus has high transcription levels of YAP and maintains high tension through FA sensing and conducting force. Meanwhile, although the FA length of F‐3Y‐TZP and F‐PEKK are similar to that of FCBIC, the transcription of YAP and the tension in the nucleus are lower. These phenomena coincide to the Figure 4d, and verify the inherent mechanisms of the optical osteoinductive effect of FCBIC.

### 2.4 In Vivo Ossification and Osseointegration Verification Experiments

**Figure 5:**
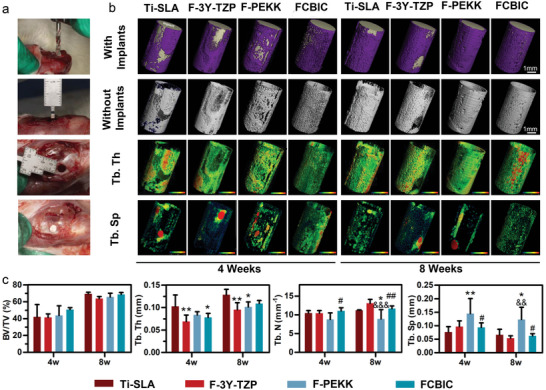
µCT evaluation of osseointegration on implants 4 and 8 weeks post‐operation. a) Digital images of the implant surgery. b) 3D reconstructed µCT images of newly regenerated bone around the implants. c) Quantitative analysis of µCT data (*n* = 4). *p < 0.05, **p < 0.01 versus sandblasted, large‐grit, acid‐etched Ti (Ti‐SLA); ^&&^ *p* < 0.01, ^&&&^ *p* < 0.001 versus F‐3Y‐TZP; ^#^ *p* < 0.05, ^##^ *p* < 0.01 versus F‐PEKK.

High‐quality osseointegration is necessary to reduce fretting wear. Encouraged by the desired cell adhesion behavior on FCBIC, adequate cellular traction force, and osteoinductive potential of FCBIC, we assessed the ossification and osseointegration between the materials and cortical bone by establishing a cylindrical bone defect model on the tibias of rats (**Figure** **5**a). The rats were sacrificed 4 and 8 weeks after the surgery, and the tibias were harvested for µCT, histomorphometric, and immunohistochemistry analysis.

At 4 weeks post‐surgery, the µCT reconstructed 3D images visually show newly formed thin bone layers surrounding all groups of implants (Figure 5b). All groups have comparable bone volume/tissue volume, indicating the similar ossification (Figure 5c). F‐3Y‐TZP and FCBIC have the lowest trabecular thickness (Tb. Th) of 0.07 ± 0.01 mm, and 0.08 ± 0.01 mm, respectively (Figure 5d). FCBIC has the highest trabecular number (Tb. N), 11.08 ± 0.82, corresponding to the best in vitro osteoinductive effect of FCBIC (Figure 5e). F‐PEKK has the highest Tb. Sp (trabecular separation) among all groups, indicating the poor ossification (Figure 5f). At 8 weeks possurgery, implants showed a comparable bone volume/tissue volume, and visually exhibited thicker surrounding bone tissues (Figure 5b,c). Ti‐SLA, the gold‐standard implant material, has the highest Tb. Th and Tb. N, and the lowest Tb. Sp, highlighting the high‐quality ossification. Simultaneously, FCBIC has no noteworthy difference with Ti‐SLA, and has a higher Tb. N and lower Tb. Sp than F‐PEKK. These findings demonstrate that FCBIC has as excellent an osteoinductive effect in vivo as Ti‐SLA. F‐3Y‐TZP also has a moderate osteoinductive effect in vivo, whereas F‐PEKK has the poorest effect (Figure 5b–f). These phenomena, slightly different from the in vitro results, may be due to the more complex pathophysiological environment in vivo.

**Figure 6:**
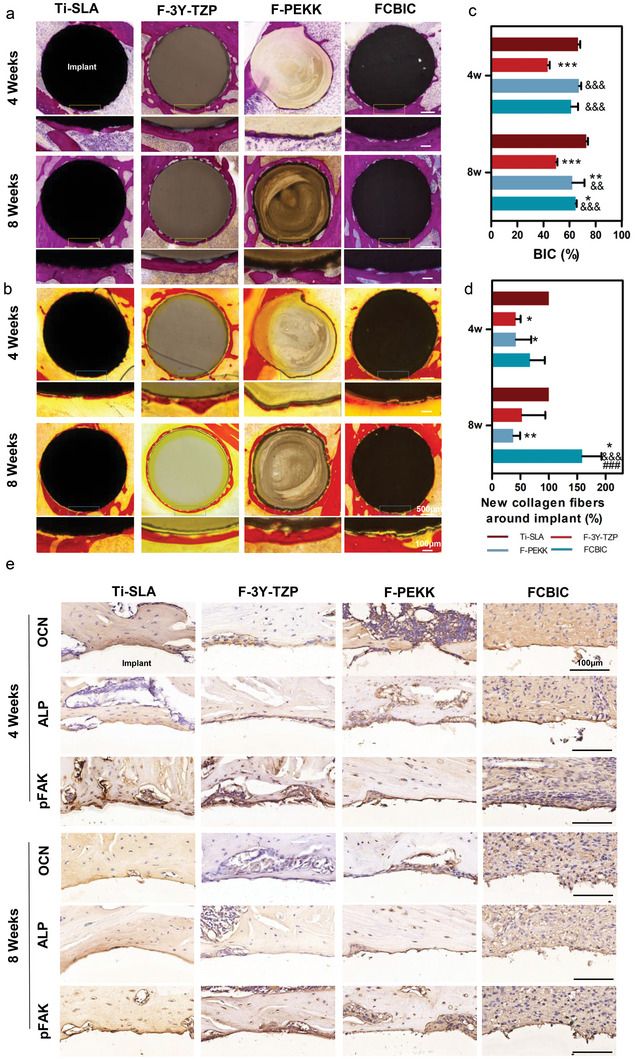
Histomorphometric and immunohistochemical analysis of the osteogenesis of the peri‐implants at 4‐ and 8‐weeks post‐operation. a) Methylene blue‐acid magenta staining and c) bone‐to‐implant contact (BIC) ratios. b) Van Gieson's picrofuchsin and d) semi‐quantitative analysis of the newly formed collagen fibers around the implant (*n* = 3). e) Immunohistochemical staining of osteocalcin (OCN), alkaline phosphatase (ALP), and phosphorylated FAK (pFAK) around implants. *p < 0.05, **p < 0.01, ***p < 0.001 versus sandblasted, large‐grit, acid‐etched Ti (Ti‐SLA); ^&&^ *p* < 0.01, ^&&&^ *p* < 0.001 versus F‐3Y‐TZP; ^###^ *p* < 0.001 versus F‐PEKK.

We comprehensively evaluated the in vivo osseointegration ability of dental materials by assessing newly formed bone and collagen fibers around implants using methylene blue–acid magenta staining and Van Gieson's picrofuchsin respectively. At 4 weeks, Ti‐SLA and F‐PEKK have the highest bone‐to‐implant contact (BIC) ratios of 66.58% and 61.24%, respectively. Collagen fibers are also generated mostly around Ti‐SLA and FCBIC. At 8 weeks, Ti‐SLA has the highest BIC ratio of 72.97%, and FCBIC has the most collagen fibers around it (**Figure** **6**a–d). These results indicate that the FCBIC group generated the comparable (4 weeks) and even more (8 weeks) collagen fibers than the Ti‐SLA group, which maintained the best in vivo osseointegration ability (Figure 6a–d). This may be because of the superior inherent osseointegration ability of Ti‐SLA. According to the analysis of cellular mechanosensing in Section 2.3, we verified these results in vivo to visually investigate the synergistic effect between the mechanotransduction and osteogenesis during osseointegration. Immunohistochemical staining was conducted for in situ analysis of the quantity of osteogenesis‐associated and mechanotransduction proteins (Figure 6e). As illustrated above, mechanosensing is one of the key mediators. The level of phosphorylated FAK (pFAK) can reflect the cellular contractility and nuclear tension generated on the surface of materials.^[^ ^42^ ^]^ Cellular contractility can promote osteogenesis, which was evaluated using mature‐stage bone marker, osteocalcin (OCN) that acts in the bone matrix to regulate mineralization.^[^ ^44^ ^]^ The expression of ALP can reflect the osteogenic differentiation of the specific area. The positive labeling for pFAK, OCN, and ALP was abundant and continuous around Ti‐SLA and FCBIC at 4‐ and 8‐ weeks post‐surgery. In the F‐3Y‐TZP and F‐PEKK groups, pFAK, OCN, and ALP also accumulated in areas without osseointegration, contributing to ossification. Overall, FCBIC provided a conductive osteogenic microenvironment for MSCs in vivo, but exhibits a lower BIC ratio than that of Ti‐SLA, which has the highest BIC ratio.

### 2.5 Tangential Fretting Features

**Figure 7:**
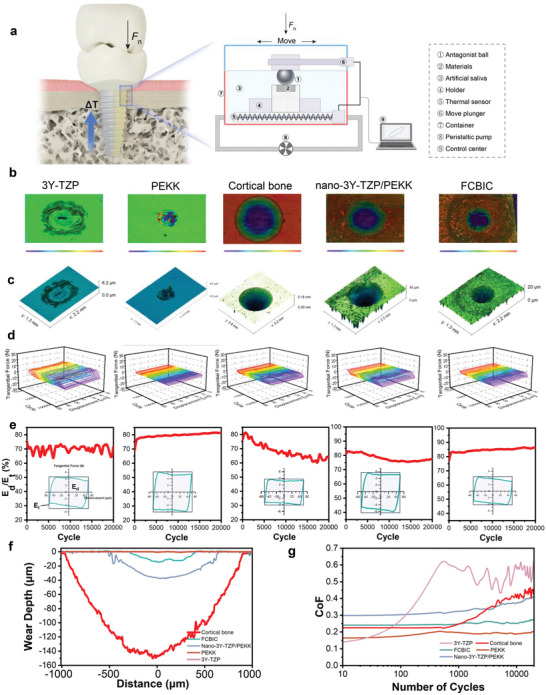
Tangential fretting features. a) Schematic diagram of tangential fretting in the dental implant/cortical bone interface and fretting test on materials. b) 2D and c) 3D maps of wear scars (*D* = 50 µm), and d) the corresponding friction log (*F*_t_–*D*–*N* curves). e) Energy ratio (*E*_d_/*E*_t_) analysis. f) The depth profiles of wear scars. g) The coefficient of friction (CoF) of the materials.

Osseointegrated dental implants experience countless masticatory motions, and therefore, fretting wear is unavoidable and irreversible between the implant and the marginal bone. Existing studies have found that marginal bone loss induced by fretting wear is one of the most common implant complications, and biomechanical impact is critical for treament success.^[^ ^45^ ^]^ Thus, tangential fretting at the main contacting interface in the cervical region of the implant should be avoided.^[^ ^9^ ^]^ We evaluated the tangential fretting wear behavior of FCBIC and cortical bone and compared it with that of commonly used implant materials by simplifying it to a titanium ball‐on‐material flat configuration in vitro (**Figure** **7**a). The displacement (*D*) was set to 50 µm, and imposed load (*F*_n_) was set to 20 N to simulate the micromotion of mastication. The frequency was set to 2 Hz to simulate human mastication frequency.^[^ ^46^ ^]^

The 2D and 3D topography of the wear scars are presented in Figure 7b,c. All the wear scars are ellipse‐shaped, as formed by the micromotion of the antagonist ball. The 3D view of tangential force–displacement–circles curves (*F*_t_–*D*–*N* curves) is depicted in Figure 7d. Using the *F*_t_–*D*–*N* curves, the energy ratio (*E*_d_/*E*_t_) for all friction pairs was easily obtained. For all friction pairs, the *F*_t_–*D* curves all present a parallelogram and the corresponding energy ratio also exceeds 0.2 during the entire fretting process, indicating that all groups are under the gross slip condition. The larger the extent of the *F*_t_–*D*–*N* curves opening, the greater the gross slip. The PEKK can help to effectively reduce the gross slip extent of composites (Figure 7d,e). The profile of wear scars and friction coefficient (CoF, the ratio of tangential force to normal force) for five materials are shown in the Figure 7f,g. It can be seen that the CoF of 3Y‐TZP is the highest at the stable stage of wear, while the width and depth of the wear scar are very small. This may be due to the high hardness of TZP ceramics, which results in a large tangential force; however, this high hardness greatly improves the wear resistance of the material (this may aggravate the wear of the matched material). On the contrary, PEKK has the lowest CoF and the lowest wear rate. This is mainly because polymer debris can play the role of lubrication and antifriction.^[^ ^7^ ^]^ In addition, owing to high elasticity of PEKK, the micro‐motion is mostly coordinated by elastic deformation. The CoF and wear volume of nano‐3Y‐TZP/PEKK and FCBIC are intermediate of 3Y‐TZP and PEKK, indicating that the addition of polymer phase reduces the tangential force in fretting and play a lubricating role, thus reducing the friction coefficient and wear rate. Compared with nano‐3Y‐TZP/PEKK, the finer and more uniform support structure of FCBIC can improve the contacting state of the contact area, further reducing the tangential force during fretting and the subsequent wear loss.

### 2.6 Biological–Mechanical Adaptivity

The structural and functional connection between the cortical bone of the edentulous area and the dental implant exists as osseointegration. Unlike the periodontal ligament connection, osseointegration is vulnerable and lacks cushioning. Biology and mechanism are two essential factors, enhancing the osseointegration, and relieving stress shielding, and fretting.^[^ ^12^, ^47^ ^]^ Here, we use biological–mechanical co‐operation coefficient to determine the biological–mechanical adaptivity of materials. This was done to highlight the coupling and interaction mechanism, through the Technique for Order of Preference by Similarity to Ideal Solution (TOPSIS) of synthetical evaluation, considering different factors and obtaining a quantitative index. We define parameters *A*, *B*, and *M*, as the degree of biological–mechanical adaptivity, biological matching coefficient, and mechanical matching coefficient respectively.

Step 1: We create an evaluation matrix consisting of *m* alternatives (Ti‐SLA, F‐3Y‐TZP, F‐PEKK, FCBIC, and bone) and *n* attributes (representative factors), with the intersection of each alternative given as (*x_ij_)_m_*_×_ *_n_*. The *n* of *B* comprises the quantitative analysis of the extracellular calcium matrix, nuclear location of YAP, parameters for µCT quantitative analysis, BIC ratios, and new collagen fibers ratios. The *n* of *M* included hardness, Young's modulus, compressive modulus, and flexural strength of materials, respectively (Table S3, Supporting Information).

Step 2: Normalize the matrix(*x_ij_)_m_*_×_ *_n_* to form the matrix *R* = (*r_ij_*)*_m_*_×_ *_n_*, using the normalization method. The equation is expressed as follow:

(1) $$r_{ij}=\frac{x_{ij}}{\sqrt{\sum_{i=1}^{m}x_{ij}^{2}}},\;i=1,2,\text{…},m,\;j=1,2,\text{…},n$$

Step 3: Determine the negative ideal solution (ideal worst alternative) *Z* ^−^ and the positive ideal solution *Z* ^+^. The *Z* ^+^ of *M* comprised the cortical bone‐related parameters.

Step 4: Calculate the distance between the target alternative *x_ij_* and the ideal solutions *Z* ^+^ and *Z* ^−^.

(2) $$D_{i}^{+}=\sqrt{\sum_{j=1}^{n}{\left(x_{ij}-Z^{+}\right)}^{2}},\;i=1,2,\text{…},m,\;j=1,2,\text{…},n$$

(3) $$D_{i}^{-}=\sqrt{\sum_{j=1}^{n}{\left(x_{ij}-Z^{-}\right)}^{2}},\;i=1,2,\text{…},m,\;j=1,2,\text{…},n$$

Step 5: Calculate the similarity to the ideal solution.

(4) $$B_{i}\;or\;M_{i}=\frac{D_{i}^{-}}{D_{i}^{+}+D_{i}^{-}}$$

(5) $$A_{i}=\frac{B_{i}+M_{i}}{2}$$

0 ≤ *A_i_* ≤ 1, *i* = 1, 2, …, *m* (*A_i_* = 0 if and only if the *m* is the best condition, while *A_i_* = 0 if and only if the *m* is the worst condition).

The FCBIC has the highest *B* of 0.67, while that for Ti‐SLA is 0.49. The F‐PEKK has the highest *M* of 0.95 and FCBIC has 0.91. This is attribute to the low Young's modulus of F‐PEKK, which is close to cortical bone. However, FCBIC appropriately increase the Young's modulus of FCBIC increaded to 39.80 GPa. The degrees of biological–mechanical adaptivity of Ti‐SLA, F‐3Y‐TZP, F‐PEKK, and FCBIC were 0.58, 0.15, 0.67, and 0.79, respectively. Taking advantage of F‐3Y‐TZP and F‐PEKK, FCBIC had the highest *A* (Table S4, Supporting Information).

## 3 Conclusions

To conclude, for long‐term implant survival and success, fretting wear must be taken into consideration. Materials with mechanical and biological properties, adapting to the edentulous area, can effectively reduce fretting wear. In this study, we designed a cortical bone analog, synthesized by stiff pDA‐nano‐3Y‐TZP scaffolds and soft PEKK through in situ polymerization. We highlight the enhancement of biological–mechanical adaptivity (*A*), considering the biological and mechanical performance. Of the composites, FCBIC exhibit bone‐matching elastic modulus, hardness, strength, and elastic‐plastic feature, as well as excellent fracture toughness. In vitro investigation of the biological effects revealed that FCBIC had no significant impact on cytotoxicity. Compared with the commonly used implant materials, FCBIC show the highest extracellular calcium deposition, ALP activity, and cellular mechanosensing, which directly correlates with the enhanced osteogenic differentiation of hWJ‐MSCs. In vivo, FCBIC is as outstanding as Ti‐SLA in terms of enhancing osteogenesis and osseointegration. Furthermore, fretting wear tests certify its lubricating effect, and ability to confer protection ability to cortical bone. These observations collectively suggest that FCBIC is an ideal dental implant material that resists fretting wear by mechanical and biological coupling, and demonstrates biological–mechanical adaptivity.

## 4 Experimental Section

### Preparation of pDA‐Nano‐3Y‐TZP

20 g L^−1^ nano‐3Y‐TZP (density = 4.58 g cm^−3^, ≈40 nm in diameter, near‐spherical particles, Nanjing Emperor Nano Material Co., Ltd., Nanjing, China) and 8 g L^−1^ dopamine hydrochloride (Beijing Solabao Technology Co., Ltd., Beijing, China) were ultrasonically dispersed in tris buffer (10 mm, pH 8.5). After stirring at room temperature for 12 h, the pDA‐modified nano‐3Y‐TZP was purified by centrifugation, dried, and ground for later use, and termed as pDA‐nano‐3Y‐TZP.

### Fabrication of Environmentally Friendly Lamellar Nano‐3Y‐TZP and pDA‐3Y‐TZP Scaffolds

The lamellar scaffold was created using an environmentally friendly freeze‐casting technique, which was realized using a homemade directional freezing machine with a controllable cooling rate.^[^ ^48^ ^]^ Slurry with ceramic‐to‐water ratios in weights of 40%, 50%, 53%, 55%, and 60% was prepared by mixing nano‐3Y‐TZP (density = 4.58 g cm^−3^, particle size ≈20 × 100 nm) (Nanjing Emperor Nano Material Co., Ltd., China) or pDA‐nano‐3Y‐TZP in deionized water with 0.5 wt% hydroxyethyl cellulose (H300, Lotte Co., Ltd., Seoul, Korea), 0.75 wt% Dynol 604 (Air Products and Chemicals Inc., Allentown, PA, USA), and 2 wt% PVA 1788 (Chengdu Kelong Chemical Co., Ltd., Chengdu, China). The slurries were blended ultrasonically, ball‐milled for 3 h, and then poured into a Teflon mold with the necessary shapes, whose the bottom was a copper cold finger. The cold finger was then immersed in liquid nitrogen. The top of the cold finger had a heat detector followed by a ring heater. The heat detector registered the temperature of the mold copper base and accordingly preset a cooling rate (2.5 °C min^−1^) feedback to heat the ring. The frozen samples were freeze‐dried in a vacuum freeze dryer (SP SCIENTIFIC, Warminster, PA, USA) for 1 week, pre‐fired at 500 °C for 2 h in a Sintering Furnace (AGT/S, Aidite,Qinhuangdao, China) to remove the organics, and then, sintered at 1550 °C for 3 h to form the lamellar structures.

### Preparation of CBIC and FCBIC

CBIC was synthesized via pDA‐nano‐3Y‐TZP scaffolds in situ polymerization of PEKK. To enhance ceramic–polymer interfacial bonding, 20 wt% *γ*‐MPS (Macklin, Shanghai, China) was used for grafting the pDA‐nano‐3Y‐TZP scaffolds first, which was mixed with a solution of methanol/water (9:1 by weight, pH = 4) for 24 h, dried at 40 °C for 48 h, and then infiltrated in the reaction system of PEKK under a nitrogen atmosphere.^[^ ^7^ ^]^ Next, the composites were heat‐treated at 380 °C for 30 min under an argon atmosphere to melt the PEKK particles, to form a combination with the scaffolds. As a comparison, 53 wt% nano‐3Y‐TZP scaffolds were also used for in situ polymerization, serving as nano‐3Y‐TZP/PEKK. FCBIC was prepared by treating CBIC with 30% H_2_O_2_ for 2 min and irradiation under a high‐pressure mercury lamp (500 W, Beijing Tianmai Henghui Light Source Electric Appliance Co., Ltd., Beijing, China) to enhance biocompatibility.^[^ ^49^ ^]^

### Characterization

Characterization details of FCBIC, nano‐3Y‐TZP/PEKK, and commonly used dental implant materials are provided in the Supporting Information.

## Conflict of Interest

The authors declare no conflict of interest.

## Supporting information

Supporting Information

## Data Availability

The data that support the findings of this study are available from the corresponding author upon reasonable request.
