# Supplementary material for: Functionalized Cortical Bone‐Inspired Composites Adapt to the Mechanical and Biological Properties of the Edentulous Area to Resist Fretting Wear
Source: Adv Sci (Weinh). 2023 Feb 12;10(11):2207255. doi: 10.1002/advs.202207255 (PMC10104646; doi:10.1002/advs.202207255)
Supplement: Supplementary file 1 — Supporting Information [file ADVS-10-2207255-s001.pdf]

Supporting Information

Functionalized Cortical Bone-Inspired Composites Adapt to the Mechanical and Biological Properties of the Edentulous Area to Resist Fretting Wear

ZhongYi Wang, QianRong Xiang, Xin Tan, YaDong Zhang, HaoQi Zhu, Jian Pu, JiKui Sun, ManLin Sun, YingKai Wang, Qiang Wei, HaiYang Yu**

1. Experimental Section

1.1 In situ polymerization of PEKK

Nano-3Y-TZP scaffolds, pDA-nano-3Y-TZP scaffolds, 0.14 mol terephthaloyl chloride (TPC), 0.04 mol isophthaloyl dichloride (IPC), 0.2 mol diphenyl ether (DPE), 0.5 mol 1-Methyl-2-pyrrolidinone (NMP), and 600 mL dichloroethane were poured into a 2 L reactor equipped with an N_2 inlet and stirred until the temperature dropped to -15 °C. Next, 104 g (0.78 mol) $AlCl_3$ was slowly added to the reaction mixture and the contents of the reactor stirred for 2 h at -15 °C. Next, the temperature was raised to 22 °C and stirred for 24 h. The composites were then taken out and immersed in methanol for 2 h. Afterward, the composites were refluxed twice in methanol for 4 h, deionized water for 4 h, and dried at 150 °C for 6 h.

1.2 Material characterization

The microstructure of the scaffolds and composites perpendicular to the freezing direction were characterized using environmental scanning electron microscopy (ESEM, JSM-IT500, JEOL, Japan) after a gold coating was added to the surface of the samples. The surface constituents were characterized using energy dispersive spectroscopy (EDS, JSM-IT500, JEOL, Japan). The pore width and lamella thickness of the scaffolds were measured using the Nano Measure 1.2 software (Fudan University, Shanghai, China) based on SEM images. The morphology and distribution of the grain size of 3Y-TZP and pDA-3Y-TZP nanopowders were characterized by

transmission electron microscopy (TEM, JEM-2100Plus, Japan). X-ray diffraction (XRD) analyses were conducted using an X-ray diffractometer (D8 Advance, Bruker AXS, Germany) with a scan range of 10–80° and a scan rate of 5° min^{-1}. Thermogravimetric analysis (TGA) and differential scanning calorimetry (DSC) were conducted using a Thermogravimetric analyzer (Perkin-Elmer 7, USA) under a nitrogen atmosphere with a heating rate of 10 ℃ min^{-1}. Fourier transform infrared spectroscopy (FTIR) was performed using an infrared spectrometer (Boguang Technology Co. Ltd., Shanghai, China). The elemental groups on the surface of the material before and after functionalization were characterized using X-ray photoelectron spectroscopy (XPS, AXIS Supra, Kratos, US). Fresh bovine cortical bones in edentulous area were obtained to donated by patients who had signed an informed consent form. The FCBIC slices were cut through a focused ion beam (FIB, FEI Strata 400S, USA) and a high-angle annular dark field (HAADF) transmission electron microscopy mode (STEM) image of the sliced specimens was obtained using TEM.

1.3 Mechanical properties

Rheological experiments on 53 wt% nano-3Y-TZP and pDA-3Y-TZP slurry were performed using a high-shear rheometer (Discovery HR-20, TA instruments, USA) with 10 rad/s angular frequency at 25 ℃. Compression and tensile tests were performed using an Instron testing machine (Instron 3400; Instron, MA, US) at room temperature. Cylindrical samples were prepared for monotonic compression tests with a displacement rate of 0.5 mm min^{-1}. The cyclic compression tests were conducted with a fixed strain rate (10^{-3} s^{-1}) and a constant displacement increase, by loading and unloading samples repeatedly until the samples failed.[1] Young's moduli and hardness were measured using an indentation test using the TriboIndenter system (Hysitron Inc. TI950, Minneapolis, MN, USA) with a load of 6,000 μN and a trapezoid function including a linear loading period, a hold period at the peak load, and an unloading period for 20 s each on polished samples up to 1 μm. The flexural

strength was measured by a three-point bending test using an electro force 3310 series II test instruments (3310-AT series II, TA instruments, USA). The specimens were 3.0 mm in thickness and 4.0 mm in width. The support span was 20 mm and the bending displacement rate was 0.000083 mm s^{-1}. The flexural strength was calculated using the following equation:

$$\sigma = \frac{3Pl}{4wb^2} \#(1)$$

Where P is the breaking load, l is the length of support span, w is the specimen width, and b is the specimen thickness.

1.4 In vitro wear tests

Bovine cortical bones were used because their physical and mechanical properties are similar to that of the human cortical bone.[2] The fretting wear tests were conducted by a fretting and wear tester (FFT-MI, RETC Instrument, USA) with an amplitudes of 100 μm and a tribometer driven by a high-precision piezoelectric ceramic actuator (P-216.9s, Physik Instrumente, Germany) with an amplitude of 50 μm under artificial saliva lubrication at room temperature in ball-on-flat linear reciprocating test conditions on polished samples up to 1 μm. Antagonist ball is made of grade IV titanium (TA IV) (Baoji Tianchang Nonferrous Metal Materials Co., LTD., China). The wear scars were observed through a white light interferometer 3D surface profilometer (RTEC, USA). The frequency of the reciprocating motion of horizontal sliding was 2 Hz, the vertical load was 90 N and 20 N respectively, and 20,000 cycles.[3]

1.5 Single-edge notched beam (SENB) testing

Single-edge-bend specimens (width ca. 4.0 mm, thickness ca. 2.0 mm) containing a U-groove notch (depth ca. 1.8 mm) and a V-notch (depth ca. 0.2 mm), in which the root radius for a straight-through slot terminating is about 0.1mm, were tested on an electro force 3310 series II test instrument (3310-AT series II, TA instruments, USA) at a constant displacement rate of 0.000083 mm s^{-1} with a support span of 16 mm. The

plane strain fracture toughness, J_{IC}, which presents the crack-extension resistance under conditions of crack-tip plane-strain, was calculated by J-R curve, which was acquired by quantifying the J-integral as a function of crack extension monitored in situ in the SEM. The J-integral, which characterizes the local stress-strain field around the crack front, can be obtained for pure bending as follows:

$$J = \frac{1.9A}{Bb_0} \quad (2)$$

Where A is the area under the force versus displacement record, B is the specimen thickness, and b_0 is the distance from the V-notch front to the back edge of the specimen.

The fracture toughness based on stress intensity factor, K, can be calculated using the following equation:

$$K = \sqrt{J \times \frac{E}{1 - \nu^2}} \quad \#(3)$$

where E is the Young's modulus and ν is the Poisson's ratio.

1.6 Surface and cellular properties

1.6.1 Specimen preparation

Sandblasted, large-grit, acid-etched Ti (Ti-SLA) was purchased from Puchuan Biomaterials Co. Ltd. (Chengdu, China). 3Y-TZP, PEKK, and CBIC were polished with silicon carbide abrasive paper of grades #320, #800, and #1200 and a polishing machine (Tegramin, Struers, Denmark) at a speed of 250 rpm and a pressure of 15 N. F-3Y-TZP and F-PEKK were obtained by treated with piranha solution (H_2SO_4 : H_2O_2 = 7:1) for 2 min.[4] FCBIC were obtained by treated with 30 % H_2O_2 for 2 min. All specimens were ultrasonically cleaned and UV irradiated for 1 h.[5]

1.6.2 Surface properties

The water contact angle (WCA) was measured by a Contact Angle System (Drop Shape Analyzer-DSA100, KRÜSS, Hamburg, Germany) to evaluate the hydrophilicity of the specimens. The surface roughness was measured using a white

light interferometer 3D surface profilometer (RTEC instruments, USA) and the Gwyddion 2.30 opensource scanning probe microscopy analysis software (Czech Metrology Institute, Brno, Czech Republic).

1.6.3 Cell preparation and culture

The hWJ-MSCs were isolated from fresh human umbilical cords donated by patients who had signed an informed consent form. This experiment was approved by the Research Ethics Committee of West China Hospital of Stomatology (WCHSIRB-CT-2022-084). The cells were cultured in T25 flasks (Nest biotechnology, Rahway, USA) with Dulbecco's Modified Eagle Medium/Nutrient Mixture F-12 (DMEM/F12; GIBCO, Thermo Fisher Scientific, Waltham, MA, USA) supplemented with 10 % (w/v) fetal bovine serum (FBS; GIBCO) and 1 % (w/v) penicillin and streptomycin (PS; Hyclone, GE Healthcare Life Sciences, Pasching, Austria) at 37 ℃ in an atmosphere of 5 % CO_2. The culture medium was replaced every 3 days. The cells were then detached from the vessel, collected, and centrifuged at 1,000 rpm for 5 min.

1.6.4 Cell viability assay (CCK-8 assay)

The hWJ-MSCs were cultured on the surface of samples placed in 48-well plates for up to 24 h and 72 h, and 4 specimens of each group were examined. Cell viability was performed using a Cell Counting Kit-8 (CCK-8; Dojindo, Japan). 30 μL CCK-8 solution was added to each well according to the manufacturer's instruction and the 48-well plate was incubated at 37 ℃ for 3 h. The absorbance value of each well was read at wavelength 450 nm in a microplate reader (Thermo Scientific, USA) and repeated three times.

1.7 Osteogenic properties

1.7.1 Alizarin Red S (ARS) and alkaline phosphatase (ALP) staining

The hWJ-MSCs were cultured on the surface of the samples placed in 48-well plates with osteogenic medium supplemented with osteogenic induction fluid, containing 10 mM β-glycerophosphate, 50 μg mL^{-1} L-ascorbic acid-2-phosphate, and 0.1 μM dexamethasone (Sigma-Aldrich, St Louis, MO, USA) for up to 7 and 14 days. Extracellular matrix mineralization was observed by gradient dehydration of the samples with ethanol, followed by staining in 1 % (w/v) ARS (pH 4.2) (Leagene, Leagene Biotechnology, Beijing, China), and washing with double-distilled water (twice). Next, 300 μL of 10 % (w/v) cetylpyridinium chloride monohydrate (BOMEI, BOMEI Biotechnology, Hefei, China) was added to each well, and the samples incubated for 30 min for quantitative analysis of the mineralization process. Next, 90 μL from each sample was transferred to a 96-well plate, and the absorbance was then measured at 562 nm. This was repeated three times. The ALP staining was conducted using a 5-bromo-4-chloro-3-indolyl-phosphate/nitro-blue tetrazolium (BCIP/NBT) Alkaline Phosphatase Color Development Kit (Beyotime Biotechnology, Shanghai, China). Briefly, the samples were incubated in 4 % paraformaldehyde and then stained in a BCIP/NBT for 4 h and photographed. This was repeated three times.

1.7.2 Cell morphology

The morphology of hWJ-MSCs for each group was observed by SEM (JSM-IT500, JEOL, Japan) after the cells were cultured in 48-well plates up to 24 h. The cells were gradient dehydrated with ethanol and air-dried, and then metalized with gold.

1.7.3 Immunofluorescence staining

The expression of Paxillin, Yes-Associated Protein (YAP), DAPI, and Actin were detected by immunofluorescence staining. The hWJ-MSCs were washed once with cell culture medium and twice with phosphate-buffered saline (PBS) and fixed in 4 % paraformaldehyde (PFA) solution for 30 min. The cells were permeabilized with 0.25 % (v/v) Triton-X 100 in PBS for 10 min after washing with ice-cold PBS and then washed three times with PBS. Next, the cells were blocked for 60 min in 1 % (w/v)

BSA in PBS-T (0.1 % (v/v) Triton-X 100 in PBS) and incubated with primary antibodies at 4 °C overnight after being washed briefly with PBS-T. After being rinsed twice with PBS-T and three times with PBS, the cells were incubated with the secondary antibody, DAPI, and Phalloidin (Thermo Fisher Scientific, USA) for 1 h at room temperature. The staining of the cells was visualized by 3D Imaging using Confocal Microscopy.

1.7.4 Preparation of functionalized implants

All specimens (Ti-SLA, F-3Y-TZP, F-PEKK, and FCBIC) were 3.0 mm in diameter and 5.0 mm in height. The Ti-SLA implants were purchased from Puchuan Biomaterials Co. Ltd. (Chengdu, China) and the PEKK implants were manufactured by the Suzhou Chinese Academy of Sciences by injecting molding technology with a custom model. The 3Y-TZP implants were designed using CAD software (Magics 24.0; Materialize, Leuven, Belgium) and manufactured using a zirconia disk (Saici, Aidite, China). The F-3Y-TZP, F-PEKK, and FCBIC implants were treated according to the procedure described in *1.6.1*.

1.7.5 Surgical procedure

The animal experiments were performed according to the protocols approved by the Research Ethics Committee of West China Hospital of Stomatology (WCHSIRB-CT-2022-084). Eleven weeks old healthy male Sprague Dawley (SD) rats were purchased from Chengdu Dashuo Experimental Animal Center and fed in a specific pathogen-free (SPF) animal facility with food and water *ad libitum*. A daily 12 h light/dark cycle was used to establish a bone defects mold. Thirty-two rats were randomly assigned to four groups: Ti-SLA, F-3Y-TZP, F-PEKK, and FCBIC. After anesthesia with sodium pentobarbital, a 10 mm incision on the inside of the knee joint was made to expose the femoral condyles and tibias below the knee joint. A pilot drill of a dental handpiece with a diameter of 2.8 mm was used to create the implant cavity. Next, the implants were gently implanted into the drilled holes and the soft tissues

were sutured. The measuring-and-guiding kit for implants (Chuangying Medical Instrument Co. Ltd., Jiangsu, China) was used to precisely control the implantation depth (Figure 7a). To prevent infection after surgery, iodophor was used to clean the wound and penicillin (100,000 IU) was used for intramuscular injection over the following 3 days. Four and eight weeks after surgery, the rats were euthanized after an overdose of pentobarbital. The tibia containing implants were harvested and fixed in 4 % PFA solution.

1.7.6 μCT analysis

Femurs with implants were imaged with a μCT device (vivaCT80, SCANCO Medical AG, Switzerland) at an isotropic voxel size of 12 μm, an energy setting of 70 kVp, and an integration time of 300 ms. Images were reconstructed into serial cross-sections and analyzed with the SCANCO Medical Evaluation and Visualizer software. A ring area extending from the implant periphery was reconstructed to evaluate the osteogenic effect. Here, the BV/TV, Tb. N, Tb. Th, and Tb. Sp were calculated.

1.7.7 Histology and histomorphometry

The specimens were gradient dehydrated with ethanol after μCT analysis. Next, the specimens were embedded in a methyl methacrylate solution at 50 °C until polymerization, after being emersed in dipping solutions I, II, and III for 5 days each. After, a microtome (LeicaMicrotome, Germany) was used to cut thin sections (ca. 100 μm in thickness) along the horizontal plane of the implant. The thin sections were polished to 20–30 μm in thickness. The sections were stained with methylene blue-acid magenta. The red color represented the mineralized bone tissue.[6] Semi-quantitative analyses were conducted by calculating the percentages of positively-stained areas versus the total field areas. Photoshop software was used to measure and calculate static histomorphometry. Van Gieson's picrofuchsin was used

to distinguish the collagen and other tissues connected with implants.[7] The red color represented the collagen fibers.[8]

1.8 Statistical analysis

Numerical data are presented as the mean and standard deviation (mean ± SD). The SPSS 24.0 software (SPSS Inc., Chicago, USA) was used to analyze the differences among the groups using a one-way or two-way ANOVA. A P value < 0.05 was considered statistically significant. Statistical analysis was performed using GraphPad Prism 5.01 (GraphPad Software, La Jolla, CA).

2. Results

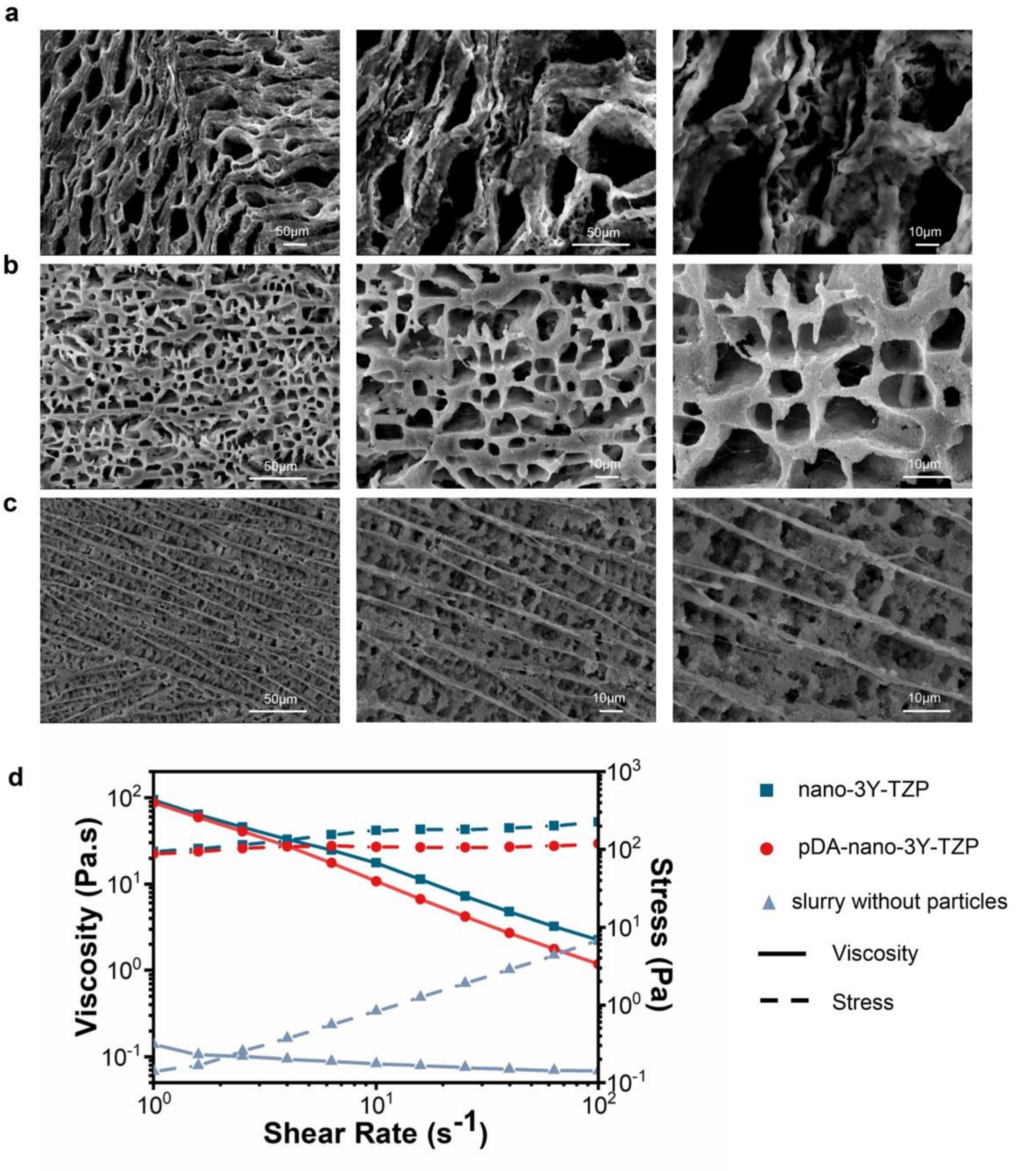

Figure S1. Representative scanning electron microscopy (SEM) images of freeze-dried unsintered a) scaffolds without particles, b) 53 wt% nano-3Y-TZP scaffolds, and c) 53 wt% pDA-nano-3Y-TZP scaffolds. d) Viscosity and stress of nano-3Y-TZP slurry, pDA-nano-3Y-TZP slurry, and slurry without particles.

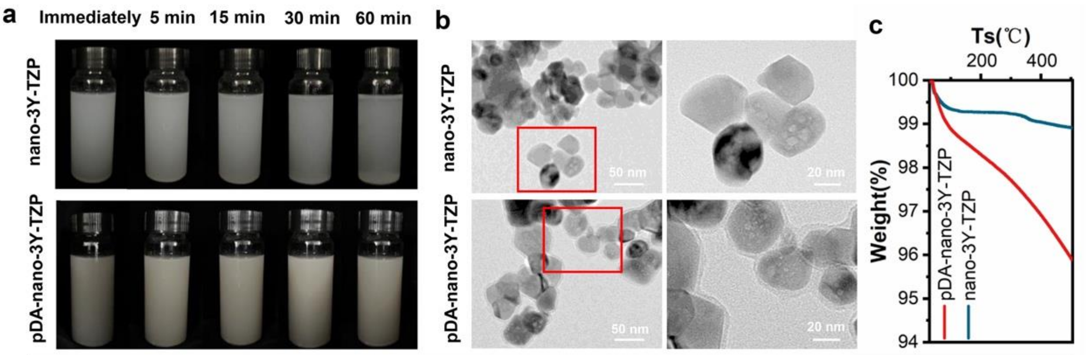

d

	Form	Primary size (TEM)	Hydrodynamic diameter (nm)	Zeta potential (mv)
nano-3Y-TZP	near-spherical nanopowder	~40 nm	209.4 ± 0.4	9.8 ± 0.5
pDA-nano-3Y-TZP	near-spherical nanopowder	~45 nm	251.3 ± 5.1**	- 8.6 ± 0.5***

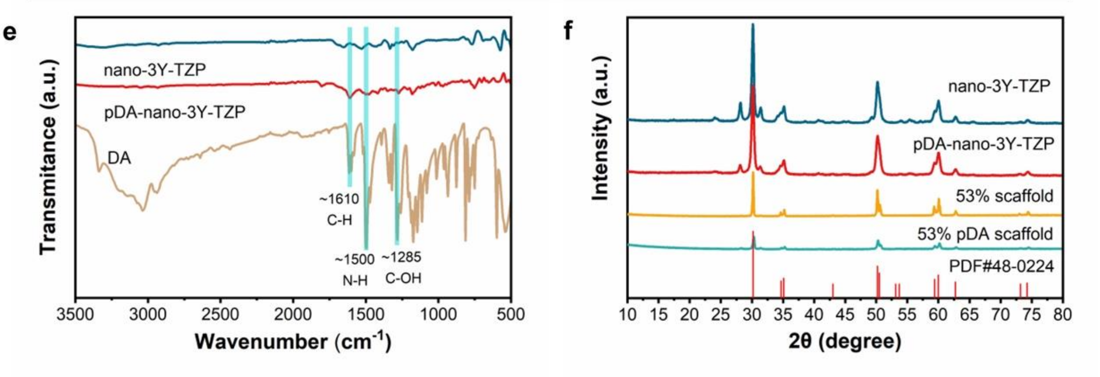

Figure S2. Characterization of nano-3Y-TZP and pDA-nano-3Y-TZP particles. a) Particle dispersibility in ultrapure water. b) Transmission electron microscopy (TEM) micrographs, c) thermogravimetric curves, d) hydrodynamic size and zeta potential of particles. e) Fourier Transform Infrared Spectroscopy (FTIR) spectra of particles and dopamine (DA). f) X-ray diffraction (XRD) patterns of particles, 53 wt% nano-3Y-TZP scaffolds, and 53 wt% pDA-nano-3Y-TZP scaffolds.

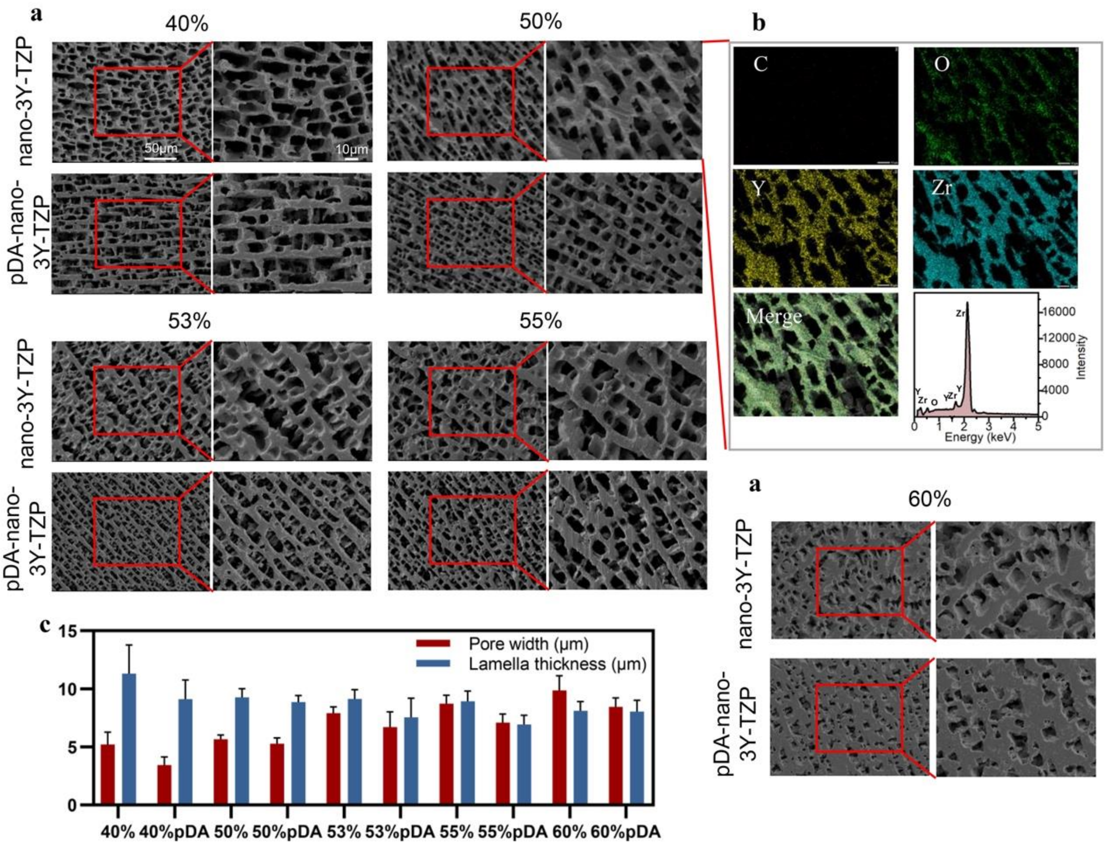

Figure S3. Cross-sectional scanning electron microscopy (SEM) images of a) the lamellar nano-3Y-TZP and pDA-nano-3Y-TZP scaffolds perpendicular to the freezing direction, with regard to c) pore width and lamella thickness. b) Energy dispersive spectroscopy (EDS) images of 50 wt% nano-3Y-TZP scaffolds.

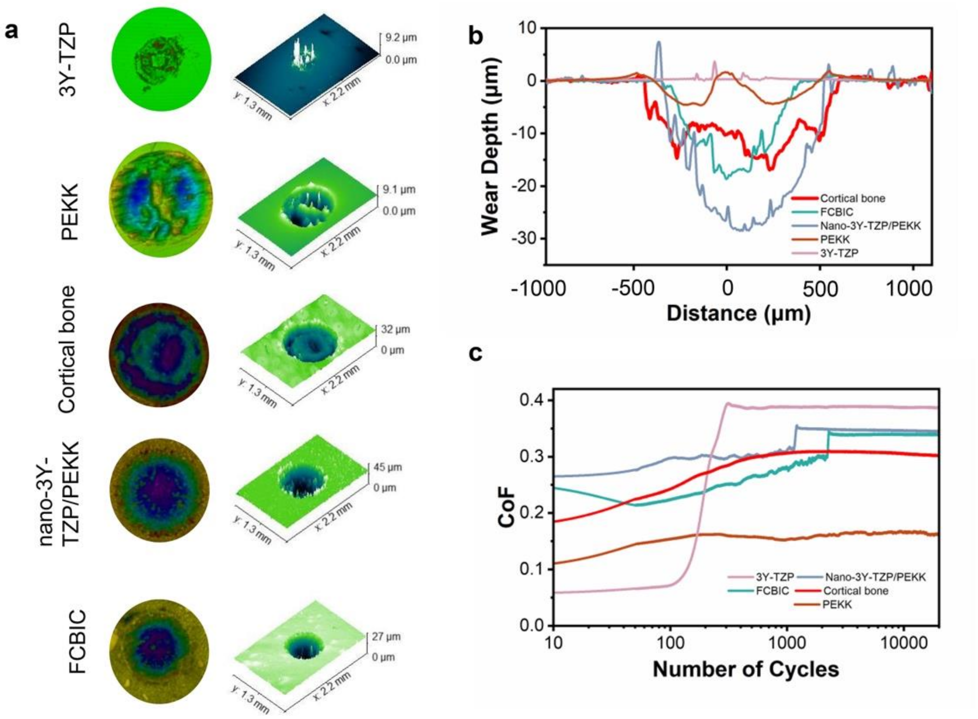

Figure S4. Tangential fretting features. a) The two-dimensional (2D) and 3D maps of wear scars (D = 100 μm, Fn = 90N, Hz = 2). b) The depth profiles of wear scars. c) The coefficient of friction (CoF) of the materials.

Table S1. Pore width and lamella thickness of the lamellar nano-3Y-TZP and pDA-nano-3Y-TZP scaffolds with different particle contents, perpendicular to the freezing direction.

		Pore width (μm)	Lamella thickness (μm)
40 wt%	nano-3Y-TZP	11.31 ± 2.48	5.21 ± 1.06
	pDA-nano-3Y-TZP	9.11 ± 1.65	3.43 ± 0.72
50 wt%	nano-3Y-TZP	9.28 ± 0.74	5.67 ± 0.36
	pDA-nano-3Y-TZP	8.86 ± 0.58	5.28 ± 0.50
53 wt%	nano-3Y-TZP	9.14 ± 0.78	7.91 ± 0.54
	pDA-nano-3Y-TZP	7.55 ± 1.65	6.70 ± 1.32
55 wt%	nano-3Y-TZP	8.94 ± 0.87	8.72 ± 0.73
	pDA-nano-3Y-TZP	6.92 ± 0.79	7.10 ± 0.75

60 wt%	nano-3Y-TZP	8.12 ± 0.80	9.86 ± 1.28
	pDA-nano-3Y-TZP	8.04 ± 0.97	8.46 ± 0.76

Table S2. Compressive strength and modulus of the nano-3Y-TZP/PEKK and pDA-nano-3Y-TZP/PEKK composites with different nano-3Y-TZP/pDA-nano-3Y-TZP/PEKK contents, compared to the lamellar scaffolds.

		nano-3Y-TZP/PEKK		pDA-nano-3Y-TZP/PEKK	
		Compressive strength (Mpa)	Compressive modulus (Gpa)	Compressive strength (Mpa)	Compressive modulus (Gpa)
40 wt%	Scaffold	67.63 ± 12.83	2.68 ± 0.64	72.32 ± 39.96	3.91 ± 2.49
	Composites	68.44 ± 9.35	4.40 ± 1.00	106.32 ± 57.20	5.23 ± 1.06
50 wt%	Scaffold	134.25 ± 21.35	4.55 ± 0.98	144.47 ± 22.12	5.03 ± 1.34
	Composites	165.33 ± 20.97	6.23 ± 2.97	183.25 ± 31.98	9.30 ± 3.05
53 wt%	Scaffold	168.84 ± 5.27	6.03 ± 1.16	290.18 ± 30.17	11.10 ± 2.36
	Composites	362.15 ± 51.56	15.50 ± 3.49	500.27 ± 85.17	24.16 ± 0.99
55 wt%	Scaffold	193.83 ± 34.45	8.12 ± 3.21	398.58 ± 160.13	12.19 ± 4.31
	Composites	189.50 ± 35.92	9.00 ± 2.69	565.70 ± 150.19	14.80 ± 1.28
60 wt%	Scaffold	215.98 ± 39.33	9.51 ± 1.65	377.16 ± 138.53	10.70 ± 2.77
	Composites	201.93 ± 44.17	8.18 ± 1.84	466.02 ± 48.57	14.45 ± 1.58

Table S3. The *m* and *n* of biological matching coefficient (*B*) and mechanical matching coefficient (*M*).

	n									
B		Alizarin Red S		Cellular Mechanosensing	μCT				Histomorphometric analysis	
	m	7d	14d	nucleus location of	BV/TV	TH	N	SP	BIC	Collagen

				YAP						fibers
	Ti-SLA	0.35	0.59	4.61	0.69	0.13	11.21	0.07	0.73	1.00
	F-3Y-TZP	0.24	0.58	4.29	0.64	0.10	13.10	0.05	0.50	0.53
	F-PEKK	0.36	0.76	2.05	0.66	0.10	8.90	0.12	0.62	0.38
	FCBIC	0.46	1.03	3.95	0.69	0.11	11.66	0.06	0.65	1.59
		n								
M	*m*	Hardness	Young's modulus	Compression modulus	Flexural strength					
	Ti-SLA	2.97	100.00	90.00	380.00					
	F-3Y-TZP	13.67	178.50	233.00	1000.00					
	F-PEKK	0.32	5.34	5.10	110.00					
	FCBIC	1.64	39.80	25.00	171.10					
	Cortical bone	0.46	15.00	20.00	180.00					

Table S4. The degree of biological matching coefficient (*B*), mechanical matching coefficient (*M*) and biological-mechanical adaptivity **(***A***).**

m	Biological matching coefficient (*B*)	Mechanical matching coefficient (*M*)	Biological-mechanical compatibility **(***A***)**
Ti-SLA	0.49	0.68	0.58
F-3Y-TZP	0.29	0.00	0.15

F-PEKK	0.39	0.95	0.67
FCBIC	0.67	0.91	0.79

References

[1] G. Tan, J. Zhang, L. Zheng, D. Jiao, Z. Liu, Z. Zhang, R. O. Ritchie, *Adv. Mater.* **2019**, 31, 1904603.
[2] C. Wang, G. Zhang, Z. Li, X. Zeng, Y. Xu, S. Zhao, H. Hu, Y. Zhang, T. Ren, *Journal of the Mechanical Behavior of Biomedical Materials* **2019**, 90, 460.
[3] P. Yu, Z. Xu, D. D. Arola, J. Min, P. Zhao, S. Gao, *J MECH BEHAV BIOMED* **2017**, 74, 154.
[4] a)F. S. F. Dos Santos, M. Vieira, H. N. da Silva, H. Tomas, M. V. L. Fook, *Biomolecules* **2021**, 11; b)R. Zandparsa, N. A. Talua, M. D. Finkelman, S. E. Schaus, *J Prosthodont* **2014**, 23, 117.
[5] A. Noro, M. Kaneko, I. Murata, M. Yoshinari, *J Biomed Mater Res B Appl Biomater* **2013**, 101, 355.
[6] J. Fu, W. Zhu, X. Liu, C. Liang, Y. Zheng, Z. Li, Y. Liang, D. Zheng, S. Zhu, Z. Cui, S. Wu, *Nat Commun* **2021**, 12, 6907.
[7] J. Huang, X. Zhang, W. Yan, Z. Chen, X. Shuai, A. Wang, Y. J. N. n. Wang, Biology, medicine, **2017**, 13, 1913.
[8] S. Naraginti, P. L. Kumari, R. K. Das, A. Sivakumar, S. H. Patil, V. V. Andhalkar, *Materials Science and Engineering: C* **2016**, 62, 293.
